# ON VASCULAR STENOSIS, RESTENOSIS AND MANNOSE BINDING LECTIN

**DOI:** 10.1590/0102-6720201600010015

**Published:** 2016

**Authors:** Barbara Stadler KAHLOW, Rodrigo Araldi NERY, Thelma L SKARE, Carmen Australia Paredes Marcondes RIBAS, Gabriela Piovezani RAMOS, Roberta Dombroski PETISCO

**Affiliations:** Postgraduate Program in Principles of Surgery, Evangelic Faculty of Paraná/University Evangelic Hospital of Curitiba/Medical Research Institute, Curitiba, PR, Brazil

**Keywords:** Ischemia, Mannose binding lectin, Complement

## Abstract

Mannose binding lectin is a lectin instrumental in the innate immunity. It recognizes carbohydrate patterns found on the surface of a large number of pathogenic micro-organisms, activating the complement system. However, this protein seems to increase the tissue damage after ischemia. In this paper is reviewed some aspects of harmful role of the mannose binding lectin in ischemia/reperfusion injury.

## Abstract (pt)

Lectina de ligação à manose é uma lectina instrumental na imunidade inata. Ela reconhece padrões de hidratos de carbono encontrados na superfície de um grande número de microrganismos patogênicos, que ativam o sistema complemento. No entanto, esta proteína parece aumentar o dano tecidual após isquemia. Neste trabalho são revisados alguns aspectos do papel nocivo da lectina de ligação à manose na lesão de isquemia/reperfusão.

## INTRODUCTION

A terosclerosis is one of the leading causes of death in modern society^6^. This disease has required a big effort from all medical community not only in preventive measurements but also in the development of new drugs for treatment and procedures of revascularization in order to avoid the ischemic complications. 

Aterosclerosis is a disease condition that begins with focal thickening of the intima with accumulation of lipid-laden macrophages (or foam cells). Smooth muscle cells populate the intima and lipids accumulate both intracellularly and extracellularly, producing the fatty streak that is the nidus of an atherosclerotic plaque^6^. There is a local chronic inflammatory response promoted by high level of oxidized low-density lipoprotein that attracts a large amount of macrophages and T lymphocytes^6^^,^ 36. Soft plaques may suddenly rupture causing the development of a thrombus and incidentally leading to death of the tissues (infarction)^4^. As a player in the inflammatory response, the complement system is thought to be involved in this process of inflammation^25^. Indeed, complement activation products have been demonstrated in atherosclerotic plaques^25^.

Stents and angioplasties may be used to reduce the incidence of clinical events. Coronary stenting, for example, has become a widely accepted technique^27^. In spite of some relevant strong points favoring its use such as greater success rate and the repeatability of the procedure, several major drawbacks still persist, including restenosis within the treated vessel 1 ^,^ 10 ^,^ 19 ^,^ 20 ^,^ 28. The restenosis phenomenon is currently the object of intensive research in different areas of biomedical field and therapy. Restenosis means the reoccurrence of stenosis and it can be defined as a reduction in the circumference of the arterial lumen of 50% or more, with the majority of patients needing further angioplasty within six months^9^^,^ 28. It is due mainly to neointima formation, which is caused primarily by smooth muscular cells proliferation and migration^12^. The formation of some neointima is necessary for vessel healing after stenting but excessive neointima formation narrows its lumen and has deleterious effect^33^. Contributing factors for restenosis are local ischemia/reperfusion, alterations of shear stress of blood stream, levels of C reactive protein and homocysteine, and immune factors, among others^9^. It is a destructive event occurring as postoperative complications after angioplasty, bypass operations, or stenting. Despite the fact that restenosis differ in pathogenesis of atherosclerosis, complement system seems, again, to play an active role in the process^9^.

This review was focused on the role of mannose binding lectin, a component of complement pathway from the immune innate system, in the injury of ischemic/reperfusion syndrome and in restenosis after medical procedures. 

## MANNOSE BINDING LECTIN: DEFINITION, STRUCTURE AND BIOLOGICAL FUNCTIONS

The human complement system is comprised of three different pathways: the classical, alternative, and the more recently described lectin complement pathway^30^^,^ 31. The lectin complement pathway is an antibody-independent cascade that is normally initiated by binding of mannose-binding lectin (MBL) to cell surface carbohydrates of foreign bacteria, protozoa, or parasites^32^. After binding, MBL activates the complement system, via MBL-associated serine proteases (MASPs), initiating the complement activation^31^. So, MBL exerts an important role in the innate immune system. 

**FIGURE 1-:**
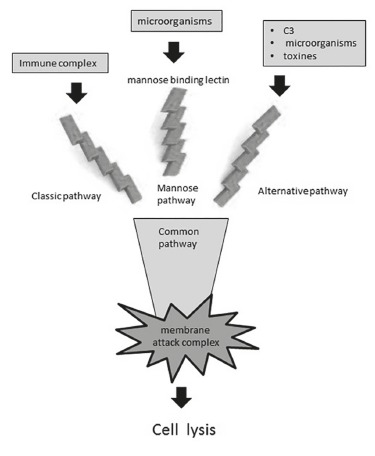
The complement system

In Figure 1, it is possible to overview the components of complement system and to locate the lectin complement pathway pathway. The activation of final pathway of complement causes the formation of the of membrane attack complex that is responsible for the destructive power of this system. Although it is intend to destroy microorganisms, the membrane attack complex may be deleterious to host cells (Figure 1).

MBL is synthesized in the liver, circulates in the serum and in inflammatory conditions can leave the circulation due to vascular leakage and be detected in the mucosal surface^14^.

Serum values of MBL may vary broadly from person to person due to the fact that the serum levels are determinate by genetic polymorphisms of the MBL2 gene, on chromosome 10 which has a high prevalence of mutations 9 ^,^ 14 ^,^ 32. High levels of MBL offer defense against invading bacteria but may be deleterious provoking local and systemic inflammation through complement activation, exacerbating inflammatory diseases, and increasing the damage resulting from ischemia and reperfusion^12^. From the evolutionary point of view, the high prevalence of mutations in the MBL gene suggests that low levels of MBL may be beneficial in some circumstances^12^.

## THE ROLE OF MBL IN ISCHEMIA AND ISCHEMIA-REPERFUSION

Several studies have implicated high MBL levels in unsuccessful outcomes in ischemic events and in clinical situations with ischemia reperfusion.

Walsh et al^35^ have done some experimental studies in mice (both wild type and Knot out for MBL) with cardiac ischemia generated by left anterior descending branch coronary artery ligation, that was loosened after 30 min. They showed that the ejection fraction of left ventricle, measured by echocardiography of wild type mice was significantly decreased. If the animal was genetically modified to be MBL null (MBL KO), the loss of left ventricular function was attenuated. Injecting MBL to MBL KO mice, higher dysfunction was again observed; if the MBL was injected together with antibodies against MBL, the dysfunction tissue injury was diminished. This experiment shows nicely the role of MBL in the ischemic/reperfusion tissue injury in cardiac tissues and raises some therapeutic possibilities. 

Gastrointestinal ischemia-reperfusion generally stems from interruption of blood flow within the superior mesenteric artery or vein and leads to small intestinal hypoperfusion and a mortality rate of 70%^29^. Clinically, it may be associated with sepsis, hemorrhagic shock, vascular surgery, small bowel transplantation 5 ^,^ 17, and multiple organ failure 16 ^,^ 24. Animal studies done by Hart et al^15^ using a mice model of intestinal ischemia/reperfusion showed that mice devoid of MBL, yet maintaining intact classical and alternative complement pathways, are protected from intestinal injury, neutrophil infiltration in the intestine, intestinal permeability dysfunction, and secondary liver injury, as measured by transaminases. 

In cerebral tissue, researches done with middle cerebral artery occlusion/reperfusion, performed in MBL-deficient and wild-type mice, had shown that infarct volumes studied by magnetic resonance and that clinical neurological deficits were smaller in MBL knockout mice than in wild type^7^. 

Taken together these experimental animal data demonstrate the potential pathophysiologic role of MBL during conditions of ischemia and reperfusion in a variety of vascular beds.

Observational studies in humans reinforce the animal findings. A study with 99 type 1 diabetes patients undergoing simultaneous pancreas-kidney transplantation showed that low pre-transplantation mannose-binding lectin levels predict superior patient and graft survival^3^. They have also demonstrated that there is an association of MBL levels >400 ng/ml with poorer graft survival and hypothesized that MBL contributes to the pathogenesis of inflammation-induced vascular damage both in the transplanted organs and in the recipient's native blood vessels^3^.

In human biopsies, MBL-depositions were observed early after transplantation of ischemically injured kidneys^8^^,^ 34. Likewise, Fiane et al 13 found considerable MBL dependent complement activation and cytokine production in patients undergoing thoracoabdominal aortic aneurysm repair with thoracoabdominal cross-clamping, a human in vivo model of ischemia-reperfusion.

In restenosis, the role of MBL has been studied by Rugonfalvi-Kiss et al^26^ observing 123 patients who underwent carotid endarterectomy and followed-up by carotid duplex scan sonography. They have shown that reoccurrence of stenosis after carotid endarterectomy had a relationship with genetically mediated high MBL serum concentration. 

The mechanisms of injury amplification of tissue injury by MBL in this context is not completely understood. Some authors postulate that MBL activation favors local thrombosis^7^. The complement and the coagulation systems cross interact at several molecular steps^2^^,^ 22. Proteins of the lectin pathway can induce thrombus formation through thrombin activation^11^^,^ 21, thus exacerbating tissue damage after ischemia/reperfusion^18^^,^ 23 ^,^ 37.

A direct toxic effect of MBL has also been advocated. Van der Pol et al^34^, using a rat model of renal ischemia, identified that after reperfusion exposure of tubular epithelial cells to circulation-derived MBL occurred internalization of MBL followed by the rapid induction of tubular epithelial cell death. This MBL-mediated tubular injury was completely independent of complement activation since attenuation of complement activation was not protective against renal ischemic reperfusion injury. These observations suggested that MBL may have a direct toxic effect on the cells and that the membrane complex attack resulting from complement activation may not be crucial to this effect.

## CONCLUSION

Although the mechanisms by which activation of complement via MBL increase the tissue injury caused by ischemia/reperfusion are not completely understood the deleterious role of this component of innate immune system on this context is clear both in humans and in animals. Specific blockade of MBL or inhibition of the lectin complement pathway may represent a therapeutically relevant strategy for the prevention of ischemic reperfusion associated damage. 
